# Tickborne Relapsing Fever, Jerusalem, Israel, 2004–2018

**DOI:** 10.3201/eid2610.181988

**Published:** 2020-10

**Authors:** Saar Hashavya, Itai Gross, Matan Gross, Noa Hurvitz, Giora Weiser, Violeta Temper, Orli Megged

**Affiliations:** Hadassah Medical Center, Jerusalem, Israel (S. Hashavya, I. Gross, M. Gross, V. Temper);; Hebrew University, Ein Kerem, Israel (N. Hurvitz);; Shaare Zedek Medical Center, Jerusalem (G. Weiser, O. Megged)

**Keywords:** tickborne relapsing fever, borreliosis, Borrelia, relapsing fever, children, adults, bacteria, Israel, vector-borne infections, ticks, spirochetes

## Abstract

To compare tickborne relapsing fever (TBRF) in children and adults in Jerusalem, Israel, we collected data from the medical records of all 92 patients with TBRF during 2004–2018. The 30 children with TBRF had more episodes of fever and lower inflammatory markers than adult patients.

Tickborne relapsing fever (TBRF), caused by *Borrelia* species bacteria, is transmitted by soft ticks of the genus *Ornithodoros* (*1*,*2*). TBRF is characterized by recurring episodes of fever often accompanied by headache, nausea, vomiting, dyspnea, and joint pain (*3*). Although highly endemic to certain regions of the world, such as West Africa (*4*), Iran (*5*), and Morocco (*6*), TBRF occurs worldwide (*7*,*8*).

In Israel, TBRF is endemic and is caused by *B. persica*, which is transmitted to humans by the *O. tholozani* soft tick (*9*,*10*). TBRF remains challenging to diagnose because of its nonspecific symptoms. To compare TBRF in children and adults, we assessed anamnestic, clinical, and laboratory parameters of persons with TBRF at 3 emergency departments (EDs) in Jerusalem, Israel.

## The Study

We reviewed the computerized databases of Hadassah Ein-Kerem Medical Center, Hadassah Mount-Scopus Medical Center, and Shaare Zedek Medical Center for all patients who had a discharge diagnosis of borreliosis (International Classification of Diseases, 10th Revision, code A69.2) during 2004–2018. These hospitals treated most patients in Jerusalem. All patients in the study with thick or thin blood film were positive for spirochetes or had positive results from a *B. persica* homemade Flagellin gene CR (*10*). For thin blood film, 1 μL was used, and for thick blood film, 10 μL. For both films, we used Giemza stain to show *Borrelia*. We completed thin and thick films for all patients with clinically suspected borreliosis. PCR was also routinely performed except for sporadic cases, for which insufficient blood remained the tube after complete blood count and films were conducted.

We defined relapse as recurrence of symptoms and positive laboratory results, after completion of treatment and without new exposures. The information collected comprised demographics (age, ethnicity, and sex); history (visits to caves, exposure to tick bites); duration of fever and number of relapses of fever; incubation period; physical examination findings; laboratory results; and hospitalization data, including length, referral to intensive care unit, and drug treatment. To determine the characteristics of TBRF in children, we compared children <18 years of age with adults.

The Hadassah medical center institutional review board approved this study and provided a consent waiver (approval no. 0345-18-HMO) We used χ^2^ tests to compare proportions and Student *t* and Mann-Whitney U tests to compare continuous nonparametric variables, and we considered p<0.05 significant. We conducted statistical analysis using SPSS Statistics 21.0 (IBM Inc., https://www.ibm.com).

Illnesses of 92 patients with blood film positive for spirochete or a positive Borrelia persica PCR met the case definition (Table). Forty (43%) patients were admitted to the hospital; the rest were discharged from the ED. The average age (± SD) was 21.3 (±10.9) years; 30 (33%) patients were children <18 years of age. Seventy-five (82%) patients were male (Figure).

**Table:** Characteristics of persons with tickborne relapsing fever, Jerusalem, Israel, 2004–2018*

Variable	Children and adolescents, n = 30	Adults, n = 62	Total, n = 92	p value
Age, y†				
Mean (95% CI)	11.65 (10.3–13.1)	25.97 (23.5–28.5)	21.3 (21.23–21.37)	<0.01
Median (range)	12 (3–18)	22.5 (19–70)	19 (3–70)	
Sex, no. (%)				
M	21 (70.0)	54 (87.1)	75 (81.5)	0.09
F	9 (30)	8 (12.9)	17 (18.5)	
Hospitalization, d				
Mean (95% CI)	2.57 (1.82–3.32)	2.66 (2.31–3.01)	2.63 (2.62–2.64)	0.39
Median (range)	2	2	2	
Mean no. ED visits (95% CI)	1.26 (1.12–1.4)	1.21 (0.99–1.43)	1.231 (1.12–1.34)	0.6
ICU admission, no. (%)	0	2 (3.2)	2 (2.2)	0.45
Treatment with doxycycline, no. (%)	25 (83.3)	61 (98.4)	86 (93.5)	0.09
Jarisch–Herxheimer reaction, no. (%)	4 (13.3)	15 (24.19)	19 (20.7)	0.35
Exposure history				
Cave visits, no. (%)	24 (80.0)	52 (83.9)	76 (82.6)	0.82
Known tick bite, no. (%)	9 (30.0)	21 (33.8)	30 (32.6)	0.88
Mean incubation period, d, (95% CI)	8.41 (6.22–10.6)	9.4 (7.06–11.74)	9.1 (9.05–9.15)	0.61
Fever				
Mean duration before ED visit, d (95% CI)	12.4 (8.53–16.27)	10 (7.31–12.7)	9.76 (9.69–9.85)	0.13
>1 Relapse of fever, no. (%)	12 (40.0)	7 (11.3)	19 (20.6)	<0.01
No. fever episodes at diagnosis				
1	6	31		<0.01
2	10	20		
3	2	4		
4	6	4		
5	4	0		
Missing information	2	3		
Signs and symptoms, no. (%)				
Gastrointestinal	16 (53.3)	22 (35.5)	38 (41.3)	0.1
Respiratory	1 (3.3)	5 (8.1)	6 (6.5)	0.39
Myalgia	8 (26.7)	22 (35.5)	30 (32.6)	0.4
Malaise	11 (36.7)	24 (38.7)	35 (38.0)	0.85
CNS symptoms	19 (63.3)	32 (51.6)	51 (55.4)	0.29
History of shivering	6 (20.0)	19 (30.6)	25 (27.2)	0.29
Organomegaly	5 (16.7)	12 (19.4)	17 (18.5)	0.76
Rash	6 (20.0)	7 (11.3)	13 (14.1)	0.26
CNS signs	4 (13.3)	2 (3.2)	6 (6.5)	0.07
Bite mark, no. (%)	11 (36.7)	17 (27.4)	28 (30.4)	0.36
Laboratory results‡				
Leukocytes, mean 10^9^/L (95% CI)	8.65 (7.71–9.59)	9.74 (7.32–12.16)	9.37 (9.35–9.39)	0.13
PMN, mean 10^9^/L (95% CI)	5.16 (4.22–6.1)	7.12 (5.34–8.89)	6.45 (9.43–6.47)	<0.01
PMN %, mean (95% CI)	0.58 (0.51–0.64)	0.72 (0.54–0.9)	0.674 (0.67–0.68)	<0.01
Lymphocytes, mean, 10^9^/L (± SD)	1.85 (1.39–2.31)	1.23 (0.92–1.54)	1.442 (1.44–1.45)	<0.01
PLT, mean 10^9^/L (95% CI)	174.2 (146–203)	136.93 (102.85–171.02)	149.64 (149.13–150.15)	0.04
PLT <150,000, no. (%)	12 (40)	35 (56.5)	47 (51.1)	0.2
Hemoglobin, mean g/dL (95% CI)	11.98 (11.3–12.7)	13.35 (10.03–16.67)	12.88 (12.87–12.89)	<0.01
CRP, median mg/dL (IQR)	7.93 (6.35–9.5)	16.87 (12.67–21.07)	12.2 (5.5–17.8)	<0.01
ESR, median mm/h (IQR)§	53.92 (40.76–67.09)	53.96 (40.53–67.39)	50 (30–75)	0.99
Hyponatremia, no. (%)	9 (30.0)	17 (27.4)	26 (28.2)	0.8
Elevated creatinine level, no. (%)¶	2 (6.7)	13 (21)	15 (16.3)	0.08
Elevated liver enzymes, no. (%)#	0	8 (12.9)	8 (8.7)	0.04

*CNS, central nervous system; CRP, C-reactive protein; ED, emergency department; ESR, erythrocyte sedimentation rate; ICU, intensive care unit; IQR, interquartile range; PLT, platelets; PMN, polymorphonuclear. †Number of persons in each group were as follows: 2–5 y: 3; 6–9 y: 5; 10–13 y: 10; 14–17 y: 12; 14–17 y: 12; 18–22 y: 31; 23–29 y: 16; 30–45 y: 10; and 46–70 y: 5. ‡Reference ranges: Platelets (10^9^/L), 150,000-400,000; C-reactive protein (mg/dL), <0.5; ESR (mm/h) <20. §Children, n = 13; adults, n = 26. ¶Elevated creatinine levels in comparison to age-adjusted reference (*11*).  #Elevated aspartate transaminase and/or alanine aminotransferase in comparison to age-adjusted reference (*11*).

**Figure:**
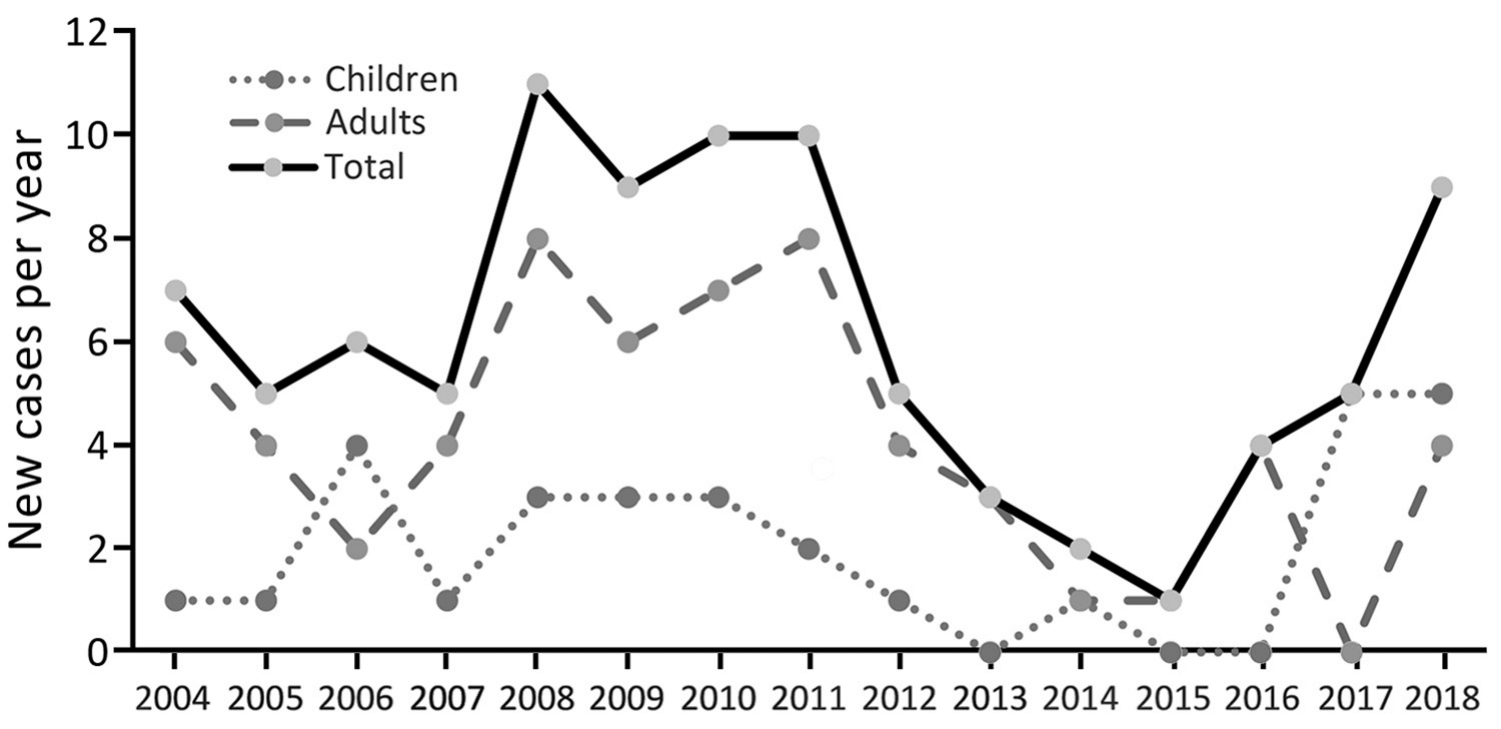
Annual number of new tickborne relapsing fever cases, Jerusalem, Israel.

Children had an average of 1.71 (95% CI 1.22–2.21) relapses of fever, whereas adults had 0.67 (95% CI 0.5–0.83) relapses (p<0.01); 40% of children had >1 relapse. The 2 groups (adults and children) did not differ significantly in terms of need for intensive care unit or hospital admission. Recent cave visits were reported for nearly 83% of patients. Although the difference was not significant, gastrointestinal symptoms (abdominal pain, vomiting, and diarrhea) occurred more often among children than adults (53.3% vs. 35.5; p = 0.1).

A tick bite mark was the most common finding on physical examination (36.7% of children vs. 27.4% of adults), followed by organomegaly (16.7% vs. 19.4%). Although the difference was not significant, neurologic signs on physical examination were more common among children than adults (13.3% vs. 3.2%).

Platelet counts were higher in children than in adults, and fewer children had thrombocytopenia (Table). C-reactive protein levels were significantly lower in children; erythrocyte sedimentation rate and total leukocyte count did not differ significantly between children and adults. The distribution of leukocyte counts differed significantly between the 2 groups: children had a lower neutrophil count and higher lymphocyte count. Children also had a lower percentage of elevated liver enzymes (0% vs. 12.9%; p = 0.04).

Twenty-six children (83%) were treated with doxycycline; 3 received azithromycin (10 mg/kg/d); and 2 received amoxicillin (50 mg/kg/d, divided into 3 daily doses). By contrast, 61 adults (98%) were treated with doxycycline (1 adult treated with ceftriaxone [2 g/day] was discharged with doxycycline). Children treated with doxycycline received 4.4 mg/kg/day, divided into 2 daily doses, and adults received 100 mg 2 times/day. One adult treated with doxycycline had 2 relapses, was re-treated with doxycycline, and recovered fully. All the patients treated with azithromycin recovered. However, illness relapsed in both children treated with amoxicillin; 1 was subsequently treated with azithromycin, and the other was treated with intravenous penicillin and intravenous ceftriaxone and discharged with azithromycin. A Jarisch-Herxheimer reaction occurred in nearly 21% of all patients (Table). All patients fully recovered.

## Conclusions

TBRF in children was characterized by more relapsing febrile episodes before medical advice was sought. One possible explanation is that febrile illnesses are more common in children than in adults, which may delay the decision to take a child to the ED or to begin a more thorough investigation in the ED. Gastrointestinal symptoms were reported more commonly in children and were the second most common symptom after fever. Findings of children from Iran who had TBRF were similar (*5*). In our study, no meningeal involvement occurred in the older group of adults; however, 1 child had suspected meningitis (21 cells in his cerebrospinal fluid with negative PCR). The expected rate is 4% in adults but is rare in children (*5*,*7*).

In adults, we found increased levels of C-reactive protein, relatively higher leukocyte counts (in reference to age norms), and higher neutrophil counts than in children. The difference in neutrophil count could be only partially explained by the difference in age-adjusted norms because only 10 children were <10 years. These findings, in addition to longer duration of fever and more relapses that did not require hospitalization, might suggest a milder course of illness in children. A possible explanation is that signs and symptoms tend to appear later in the course of the disease, and TBRF symptoms tend to be milder during relapses (*12*).

The 2 children treated with amoxicillin experienced relapses, whereas only 1 patient treated with doxycycline and none of the patients treated with azithromycin had relapse. Use of doxycycline remains controversial, despite recent reports showing its safety in children (*13*). Consistent with the literature, our findings support the safety and efficaciousness of erythromycin as an alternative treatment for children with TBRF (*5*,*14*).

This study has several limitations. Because of its retrospective design, all parameters were data retrieved from medical charts. The study’s small sample size, especially the number of children, hindered identification of other subtle differences between children and adults. Nevertheless, this study provides data on the differences between the manifestations of TBRF in children and adults.
